# The Conditions of Imperfect Development of the Septum between the Mouth and Naso-Pharynx, Usually Termed Cleft Palate*Read at the last annual meeting of the Georgia Medical Association.

**Published:** 1898-08

**Authors:** M. F. Carson

**Affiliations:** Griffin, Ga.


### THE CONDITIONS OF IMPERFECT DEVELOPMENT OF THE SEPTUM BETWEEN THE MOUTH AND NASO-PHARYNX, USUALLY TERMED CLEFT PALATE.*

By M. F. CARSON, M.D.,

GRIFFIN, GA.

In order to place their treatment on a more scientific basis, I will first consider the physiology of the nose, which means a consideration of the mechanical factors that determine the form and development of the nasal cavities of the upper portion of the pharynx, usually described together as the "naso-pharynx." We shall then be better able to formulate general principles on which our methods of treatment may be most advantageously based.

The principal function of the naso-pharynx is to transmit air into the lungs, in order that the sensitive surface of its mucous covering may become affected by substances contained in it. In short, this part of the body is constructed essentially for the purpose of smell, and in the different groups of animals the degree of its development varies with the importance of the sense of smell to them. The olfactory nerve is distributed to the upper portion of the nasal cavities, and the current of air is distributed especially over this area by the act of sniffing. During natural respiration the mouth is kept closed, the upper lip covering the upper incisor teeth entirely. During normal respiration the air passes mainly through the lower meatus, while there is practically no current in the upper portion of the cavity.

It is therefore very obvious that any obstruction sufficient to interfere with the caliber of the lower nasal cavities will eventually impair their capacity for performing the functions for which they are intended—namely, the transmission of air; and as a direct result the patient breathes through the mouth. When this is done air enters so freely through the larger and more direct channel formed by the mouth that it ceases to pass through the nose. The

Read at the last annual meeting of the Georgia Medical Association.

most important function of the part is lost, and the forces which act upon it normally to develop it are in abeyance. After air has entirely ceased to pass through the nose in respiration, it does so during the expiratory process of vocalization to a small degree. The so-called nasal intonation does not develop as early as one would imagine in association with so much loss of nasal inspiration.

It is at once visible from an examination of a vertical section through the nasal cavities that the caliber of the lower part of the cavities, or of their respiratory section, is enormously encroached upon by any ascent of the hard palate above its normal line, and that the air-transmitting capacity of the naso-pharynx varies inversely to the height of the palate. We also see that the caliber of the entire nasal space is very much affected by the slightest variation in the interval between the septum and the structures forming the outer wall of the space. We all know how exceedingly common are these cases of imperfect development of the nasal cavity. I believe that if we exclude those diseases of the nose, ear, larynx, and even lungs, which are either directly or indirectly consequent upon some imperfect development of the naso-pharynx, a not unimportant portion of the consideration and treatment of disease of these parts would have to be taken from books on this subject. It is essential, therefore, that we carefully consider the factors which are responsible for such imperfect development, in order that we may save our patients from numerous complications which may not at first sight seem directly dependent upon it. I believe that such poorly developed conditions result mainly from the fact that for some reason the mechanical factors that aid in the development of the naso-pharynx are removed, and as a result this space does not develop as it should. We should not forget the fact, however, that some children inherit a degree of development which is less than the normal. Personally, I believe that the transmission of this particular condition is very direct and rapid. The absence of this developmental factor is caused by the infection of the mucous membrane of the nose so commonly called a cold in the head. This inflammation produces swelling of the mucous membrane, which interferes sufficiently with the entry of air and renders it necessary to breathe through the mouth. This at once deprives the naso-pharynx of that factor upon which it is solely dependent for its development and growth, namely, the pressure of air upon its walls, and as a consequence it ceases to increase in size. The interval which separates the septum and the outer wall of the space remains unchanged, and the septum between the mouth and nose does not descend, the arch of the palate remainining abnormally high.

The infection of the nasal mucous membrane sets up an inflammation of the lymphatic mass in the pharynx often called the pharyngeal tonsil, and later the lymphatics of the neck, which receive lymph from these structures. The tonsils often become affected, as well as the larynx, trachea and bronchi.

The enlargement of the lymphatics of the pharynx often interferes with the normal functions of the middle ear, with which consequences we are all too familiar.

These inflamed cervical glands afford a suitable nidus for tubercular organisms, which produce such disastrous results. To this condition of nasal obstruction the word adenoids has been applied. It has become too familiar an expression, and is deservedly regarded by patients with considerable dread. I may be too young, both in years and experience, to criticise, but personally I believe that surgeons have too often regarded the secondary infection of the so-called pharyngeal tonsil as the primary cause of the naso-pharyngeal obstruction, and have endeavored to cure the condition by the unskilful and heroic scraping of these parts. It is quite apparent that this operation is not alone sufficient to restore to the nose that mechanical factor upon which its growth and development depend. Such a procedure may occasionally be of service, but the operation alone will not relieve the infected mucous membrane. On the same principle, we had as well attempt to cure a sore on the penis by removing a secondary infected bubo.

The patients who are minus the vigor and strength and energy to get rid of this primary infection by expelling the accumulated mucous, and receiving air through into the nose, are much alike in many respects. Their vital powers are *nil;* there is a total absence of energy; they always assume attitudes of rest, and avoid those of activity. The quantity of air they change during respiration is very small, and is produced mainly by the action of the diaphragm, the chest remaining in the position of rest.

If we should take the trouble to pass a tape around the chest during respiration, we will find it varies little, if at all. As a result we get the fixed resting posture of the body often described as "dorsal excurvation." Finally, as a natural result we get complete fixation, to which the popular and unscientific terms lateral curvature, scoliosis, etc., have been applied.

As a result of this resting attitude of the chest we also get other deformities called flat-foot and knock-knee. These patients are supplied with such small quantities of oxygen, and are loaded with foul pulmonary products, have little energy to expend in forcibly ventilating the naso-pharynx, but fall back on the more easy method of procuring it through the mouth, with results too familiar to all of us.

I have often wondered why it is that medical men devote so little attention to the manner in which people perform that most important function respiration, since it is by far the most important one for the welfare of the patient. By the means of this function alone are the tissues and organs supplied with oxygen; and in direct proportion as the oxygen supplied is good or bad, so are the functions of these tissues and organs performed in a satisfactory or unsatisfactory manner. The attention of most medical men has been fixed, and we have educated our patients and the general public to fix theirs, upon the rapidity with which the products of digestion pass along the intestinal canal, and it is generally assumed that something has gone wrong if the bowels fail to act as least once a day. 'Tis strange, I say, that no attention whatever is paid to the manner and character of the respiration, yet it is of infinitely more importance to the welfare of the patient. In fact, constipation is too often the result of imperfect oxygenation. I have frequently seen patients who perform little or no thoracic respiration with the spine fixed in the position of extension by some steel brace or other, and others ordered laborious exercise to rapidly exhaust the small capital without adding to it appreciably.

I believe that the erroneous treatment results from a deficient knowledge of the mechanics of respiration and of the important part which the spinal column should take in this process. Again I may be too young to criticize our able physiologists, but I believe they have misled many of us by their attempts to illustrate the respiratory process by means of a mechanism in which the spinal column is represented as a rigid and immovable rod. This generally accepted theory is absolutely false, since the variations in the spine are infinitely more visible than those of the chest-walls. I would therefore suggest that the manner in which the tissues receive their oxygen receive as much attention as the rate at which the sewage products of the body pass along the intestinal canal.

I believe, if this is done well, that adenoids, resting deformities and many other diseases resulting, either directly or indirectly, from an imperfect supply of oxygen, will disappear.

'Tis not my intention to consider the subject of adenoids further than to say that bloody operative procedures are, in my opinion, in most instances wholly unnecessary, and that a systematic ventilation of the lungs through the naso-pharynx furnishes us with a means not only of applying to the naso-pharynx that force by air being driven forcibly through it, but, by oxygenating the blood more fully, removes more thoroughly its carbonic acid gas, the tissues of the body are better nourished and perform their functions in a normal manner. In my opinion and experience, this is best accomplished by placing the child on its back three times a day for at least twenty minutes at a time, and make it breathe in and out as deeply as possible through the nose, the mouth being kept closed. This, together with some antiseptic lotion to keep the infected membrane clean, is in my judgment the most rational treatment in many diseases of the naso-pharynx.

Returning to my subject, cleft palate, you see that so long as the nasal cavities are in communication with the mouth the main factor upon which they depend for their development is held in abeyance, and therefore they cannot develop. As a consequence, the sides of the alveolar arch become closer together, as do also the edges of the cleft, and the roof becomes more vertical. Most surgeons claim that in delaying the operation these changes occur and render the operation more simple; or, to put it plainly, the least developed the nose, the easier is the closure of the fissure in the floor.

If the caliber of the nose bears an inverse proportion to the height of the palate, as the lower portion of the cavity is the portion through which the air passes, it is plain that this space is encroached upon by any increase in the height of the palate. Knowing, therefore, the mechanics of the naso-pharynx, it is easy to see the advantage of separating these two cavities as early in life as possible, in order to restore to the nose that force upon which its development depends.

In order to remind you of the practice followed by most surgeons of to-day, I will quote from three of our standard authors, both at home and abroad. Mr. Treves says that in the infant the cleft is wisely deemed inoperable, and that the time of election is from four to six years of age. The author does not appear to adduce any argument to prove that it is unwise to operate on an infant, but as he is authority we must accept it.

Mr. Erichsen says: "The first question to be determined is the age at which the operation should be performed, as the success of the operation depends mainly upon the patient remaining quiet during the operation, upon his assisting the surgeon by keeping the mouth open, and that it is not expedient to interfere until the patient is old enough to understand the necessity of keeping quiet."

Mr. Bryant says that the operation may be undertaken at the age of five in a healthy child, and that he has performed it himself successfully as early as four. Are we to accept these statements as true? When, in the past winter, in Guy's Hospital, I saw thirty-eight clefts closed, not one of them older than six weeks, and not one of them failed to recover. No. I believe that the treatment of cleft palate, like other operations in surgery, has been a creed and a tradition, and that if we treat these patients as we should no time should be lost in restoring to the nose its normal physiology.

(1) At what age should we operate?

(2) What is the best method to do it?

(3) How may complications be met?

If there is no special contraindication, I believe the best time to operate is from the fourth to sixth week. Why? First, because the child stands the operation well. Second, the child experiences very little discomfort afterwards, and is soon able to take its food. Third, the small amount of hemorrhage is easily controlled.

The details of the operation vary in different cases, but in the majority of cases we should endeavor to raise a flap of mucous membrane, together with the periosteum from one side, and attach it securely beneath the raised margin of the opposite side, a modification of Mr. Davies's college operation.

Many children with cleft palates have harelip also. The latter is best left alone till the cleft in the palate has been closed, as it affords more room to reach the cleft.

